# mSphere of Influence: Interferon Ultra Is a Means To Fight Viral Disease

**DOI:** 10.1128/mSphere.00134-20

**Published:** 2020-03-25

**Authors:** Connor G. G. Bamford

**Affiliations:** a Queen's University Belfast Wellcome-Wolfson Institute for Experimental Medicine, Belfast, United Kingdom

**Keywords:** ISG, evolution, genetics, hepatitis C virus, interferon, interferon lambda, virology, virus

## Abstract

Connor G. G. Bamford works in the fields of virology and innate immunity. In this mSphere of Influence article, he reflects on how three papers by Manry et al. (J. Manry, G. Laval, E. Patin, S. Fornarino, et al., J Exp Med 208:2747–2759, 2011, https://doi.org/10.1084/jem.20111680), Terczyńska-Dyla et al. (E. Terczyńska-Dyla, S. Bibert, F. H. T. Duong, I. Krol, et al., Nat Commun 5:5699, 2014, https://doi.org/10.1038/ncomms6699), and Schoggins et al. (J. W. Schoggins, S. J. Wilson, M. Panis, M. Y. Murphy, et al., Nature 472:481–485, 2011, https://doi.org/10.1038/nature09907) made an impact on him during a critical stage of his career and how they continue to reinforce why he researches what he does and shape the approaches he takes.

## Abstract (precis)

Connor G. G. Bamford works in the fields of virology and innate immunity. In this mSphere of Influence article, he reflects on how three papers by Manry et al. (J. Manry, G. Laval, E. Patin, S. Fornarino, et al., J Exp Med 208:2747–2759, 2011, https://doi.org/10.1084/jem.20111680), Terczyńska-Dyla et al. (E. Terczyńska-Dyla, S. Bibert, F. H. T. Duong, I. Krol, et al., Nat Commun 5:5699, 2014, https://doi.org/10.1038/ncomms6699), and Schoggins et al.

## COMMENTARY

Concerned by the encrypted Axis powers’ communications during WW2 coming from their infamous “Enigma” machines, the Allies developed the top-secret “Ultra” program to crack enemy codes and help end the fighting. Analogously, viruses are typically considered to be among humanity’s arch-nemeses. Scientists globally have been fighting for centuries via their own Ultra program, by trying to crack the code of the human immune system and of how to use it to stop viruses. One weapon we could have in our grasp is the interferon (IFN) system, consisting of three families (types 1 to 3) of antiviral cytokines that potently induce immune defense against pathogens (1). Although we have recognized the capabilities of IFNs for decades, since their discovery in 1957 by Isaacs and Lindeman (2) as a soluble viral interference factor, we are only beginning to realize their true potential in human health and disease. The papers I discuss here represent three recent examples of attempts to understand IFNs in order to fight infections.

Jérémy Manry and colleagues in 2011 (3) in Luis Quintana-Murci’s laboratory at the Institut Pasteur in Paris were interested in using the extant genetic diversity of humans and their IFN system genes (cytokines and receptors) to garner information about their function, hoping to understand their role in human survival. Before the ease of facile whole-genome sequencing, Manry and colleagues used targeted sequencing of 27 of the 28 human IFN genes (*IFNL4* had not been discovered yet) from 186 healthy people across Africa, Europe, and Asia, building the first bespoke database of IFN system genetic diversity. The Quintana-Murci lab identified over 1,000 polymorphisms and uncovered intriguing patterns in diversity that continue to fascinate and amaze us nearly a decade on, such as the loss of function of two type 1 IFNs, the high conservation of IFN alpha 8 and 14, and the action of strong positive selection in type 3 “lambda” (L) IFNs. Three years later, a study (4) from first authors Ewa Terczyńska-Dyla, Stephanie Bibert, and Francois Duong and coworkers across the labs of Rune Hartmann (Aarhus, Denmark) and the Swiss labs of Pierre-Yves Bochud (Lousanne) and Markus Heim (Basel) drilled down to the role of this coding genetic diversity in the outcomes of patients infected with chronic hepatitis C virus (HCV) infection. In the late 2000s, numerous high-profile genome-wide association studies (GWAS) (5–8) found an association between variation near type 3 IFNs and outcome of HCV infection. This phenomenon was later in 2013 (9) traced to a polymorphic pseudogene, *IFNL4*, where, paradoxically, loss of the IFN was better for you if you were infected with HCV. Terczyńska-Dyla et al. go one step further and link a common functional variant in *IFNL4* (causing a proline-to-serine mutation at position 70, P70S)—that reduces IFN-λ4 bioactivity—to outcome of HCV infection, providing the first indisputable link between IFN-λ4 biochemical changes and human disease. Finally, in 2011, John Schoggins in the lab of Charlie Rice at The Rockefeller University in New York, USA, working with their colleagues Sam Wilson and Paul Bieniasz, published (10) what would become a classic IFN study that combines elegant dual-color lentivirus-based flow cytometry assays with brute-force unbiased screening of 380 interferon-stimulated gene (ISGs) against 6 viruses to identify which ISGs are directly responsible for inhibition of a virus replication. Therein, Schoggins et al. importantly rediscovered many known antiviral ISGs and uncovered novel virus-blocking ISGs capable of stopping infections by important human pathogens, like HCV and human immunodeficiency virus.

Despite forming a strong platform arguing for the importance of the potential of IFNs, the work of Manry et al., Terczyńska-Dyla et al., and Schoggins et al. provided me with intellectual stimulus to investigate IFN biology further and demonstrated effective approaches (mostly academically rather than technologically) to address my own questions. First, they showed that understanding genetic diversity and the evolution of genes can provide critical insights into function, often in ways that could not be predicted. Second, they showed that one must ultimately investigate the mechanism of a biological phenomenon, as that allows you to apply that knowledge for human good. The studies by Terczyńska-Dyla et al. and Schoggins et al. particularly showcase the drive to mechanistically understand precisely how IFNs work. However, still—nearly a decade later—we do not yet fully comprehend how IFNs or IFN-λ4 or many ISGs really work. Finally, what all three studies share is the combination of understanding biology for its own good with understanding it for the good of humanity through helping figure out why some people are more prone to disease than others, identifying what makes an IFN contribute to health and disease, or finding new ways to stop viral infections. Most reflective of these influences is my recent paper (11) that combines human genetic diversity, antiviral activity assays, and mechanistic investigations of diversity IFN-λ4 bioactivity to understand the role of IFN-λ4 during HCV infection in humans.

Those three studies have been followed up on by publication of, to name a few, Schoggins’ characterization of the cyclic GMP-AMP synthase gene *cGAS* as an pan-antiviral factor (12), the identification of a set of “core” ISGs expressed by mammals and chickens (13); and the linking of very rare loss-of-function mutations in IFN system genes to serious outcomes in viral infection (14). In sum, the three influential studies I present here represent a source of observations to follow, demonstrate the impact that research on IFNs may have, and carve a path to follow on how to realize their potential, as part of IFN “Ultra” that fellow researchers and I have unknowingly been working on.
